# The influence of adolescents’ internet adaptation on internet addiction: the mediating role of internet cultural adaptation

**DOI:** 10.3389/fpsyt.2023.1338343

**Published:** 2024-01-08

**Authors:** Yue Yang, Jun Zhan, Yao Ni, Yanwen Fan, Yiting Zhang, Yiting Fang

**Affiliations:** ^1^School of Psychology, Zhejiang Normal University, Jinhua, China; ^2^Zhejiang Philosophy and Social Science Laboratory for the Mental Health and Crisis Intervention of Children and Adolescents, Zhejiang Normal University, Jinhua, China; ^3^Postdoctoral Station of Psychology, School of Psychology, Fujian Normal University, Fuzhou, China; ^4^Fujian Agriculture and Forestry University, Fuzhou, China

**Keywords:** internet addiction, adolescent internet use, internet cultural adaptation, internet adaptation, cross-cultural adaptation theory

## Abstract

This study investigates how adolescents’ internet adaptation influences internet addiction, with a particular focus on the mediating role of internet cultural adaptation. Grounded in cross-cultural adaptation theory, the study proposes that internet cultural adaptation can mitigate the negative relationship between internet adaptation and internet addiction. Conducting a large-scale random survey among Chinese adolescents, and employing standardized measures for internet addiction, internet cultural adaptation, and internet adaptation, the study finds a significant negative correlation between internet adaptation and internet addiction. More crucially, internet cultural adaptation plays a pivotal mediating role, such that when adolescents have higher capabilities in adapting culturally to the internet, the negative relationship between their internet adaptation and addiction is effectively alleviated. These findings not only provide a new perspective in understanding adolescent internet addiction but also offer theoretical guidance for devising preventive measures. The study also discusses practical applications of the results, emphasizing the importance of enhancing adolescents’ internet cultural adaptation, and presents new strategies for preventing and mitigating issues of internet addiction.

## 1 Introduction

In the aftermath of the pandemic, as society gradually returns to normalcy, the long-term effects of increased internet usage during the pandemic have become a focal point of academic inquiry. The pervasive shift to online platforms for work, education, and social interaction has potentially laid the groundwork for heightened susceptibility to internet addiction. Internet addiction refers to a behavioral pattern where individuals excessively rely on the internet, thereby affecting their daily life, academic performance, and interpersonal relationships (1–4). Studies suggest that this extended reliance on digital connectivity might contribute to patterns of addictive behavior, encompassing challenges in managing online engagement and its impact on mental well-being (5–7). Addressing these emergent trends is crucial in the post-pandemic landscape.

Compared with Internet addiction, a more positive results of internet use is internet adaptation, which refers to an individual’s ability to effectively manage and balance online and real-world activities, academics, and social interactions in a digital environment (8). Specifically, internet adaptation involves how one negotiates the relationship between the online world and reality, whether internet usage behaviors conform to social norms, the impact on one’s academics and life, and the rationality of internet use (9). Obviously, internet adaptation refers to an individual’s effective use of the Internet as part of their daily life and work, while internet addiction refers to excessive use of the Internet to the extent that it affects the individual’s daily life and health. It is important to note that internet adaptation may turn into internet addiction in some cases. For example, a person may initially use the internet for work or study, but overuse can lead to addiction. Therefore, studying the relationship between internet adaptation and internet addiction is critical, as it helps us better understand how individuals balance healthy and harmful Internet use. However, most relevant research has generally focused on psychosocial factors of internet addiction, the mental health effects of internet behavior, or the relationship between internet use and an individual’s ability to adapt (10, 11).

Building on the research of internet adaptation, this study introduces the concept of internet cultural adaptation. Internet cultural adaptation refers to the process and ability of individuals to adapt to and integrate into the online culture within the internet environment. According to the theory of cross-cultural adaptation, when individuals encounter a new cultural environment different from their original cultural background, they may undergo a series of psychological adjustments and adaptation processes (12). This theory can be analogously applied to the internet environment, implying that adolescents facing a diverse array of online cultures, if lacking effective adaptation strategies, might fall into what is termed “cultural shock.” This, in turn, could lead to cognitive biases, negative emotions, and even irrational online behaviors (13). As a strategy and capability for individuals to adapt in the online environment, the impact of internet culture adaptation on internet addiction and its internal mechanisms merits further investigation. Hence, this study aims to explore the relationship between internet cultural adaptation, internet adaptation, and internet addiction, particularly the mediating role of internet cultural adaptation in the relationship between internet adaptation and internet addiction.

Furthermore, this broad scope of internet engagement raises particular concerns when considering the adolescent population. Adolescents, undergoing critical phases of psychological and social development, are uniquely impacted by their digital interactions. The patterns of internet use established during this formative period can significantly shape their long-term developmental trajectory (14, 15). Given their evolving maturity and self-regulatory capabilities, adolescents are particularly vulnerable to the pitfalls of internet addiction, which could detrimentally affect their academic achievements, social skills, and overall mental well-being (5, 9, 16). Thus, a deeper exploration into the nuances of internet adaptation and cultural adaptation amongst adolescents is not only pertinent but essential for devising effective preventative and intervention strategies against internet addiction.

To this end, this study employed standardized scales for internet addiction, internet cultural adaptation, and internet adaptation, conducting a large-scale random survey among the adolescent population in China. Through an in-depth analysis of the survey data, we hope to gain a better understanding of the psychological mechanisms behind adolescent internet addiction, thereby providing a theoretical basis for developing effective prevention and intervention measures.

## 2 Methods

### 2.1 Participants

The study focuses on middle school students aged 13 to 17 nationwide. To ensure the reliability of questionnaire data, we coordinated directly with school administrators and distributed a total of 4,532 online anonymous questionnaires (male: 1,264, female: 1,857). All questionnaires were returned, but after removing invalid questionnaires, 3,121 valid responses were received, yielding a response rate of 69%.

### 2.2 Measures

#### 2.2.1 Internet addiction scale

We utilized the Internet Addiction Scale (CIAS-R), revised by Chen Shuhui et al., which was originally designed for college students in Taiwan based on the standardized Chinese Internet Addiction Scale (CIAS). This scale consists of 26 items divided into five dimensions: internet addiction tolerance, compulsive internet use, internet withdrawal reaction, interpersonal and health problems, and time management problems. Internet addiction tolerance is characterized by the need for an individual to engage with increased amounts of online content or extend their online duration in order to achieve a level of satisfaction comparable to their initial experiences. Compulsive Internet Use refers to an intense, nearly uncontrollable desire to connect online. This urge manifests when thinking about or seeing a computer, making it challenging for individuals to disengage once online. Their mental state tends to be more energized during such interactions, accompanied by a desire for prolonged online sessions. On the other hand, internet withdrawal reaction describes the emotional and psychological disturbances that occur when one is abruptly prevented from accessing the internet. Symptoms include feelings of depression, irritability, emptiness, as well as behaviors like inattention and restlessness. Additionally, overindulgence in the virtual realm can lead to interpersonal and health issues. Such immersion can cause individuals to neglect their social roles, leading to estrangement from family and friends. Concurrently, it can bring about physical discomforts, ranging from ocular issues and headaches to sleep deprivation and gastrointestinal disturbances. Lastly, time management problems arise when an individual’s excessive time online hampers their work, academic pursuits, and disrupts regular routines such as sleep and meals. The first three constitute the core symptoms of internet addiction, while the last two indicate associated issues. A 4-point scoring system was used, with 1 indicating “strongly disagree” and 4 “strongly agree.” The sum of item scores within each dimension corresponds to the total score of that dimension. The total score of all items indicates the individual’s level of internet addiction, with a higher total score implying a higher tendency for internet addiction. The scale exhibits excellent internal consistency, with Cronbach’s *α* coefficient of 0.93 for the whole scale, and 0.90 and 0.88 for the sub-scales, indicating good reliability (17).

#### 2.2.2 Adolescent internet adaptation scale

The study used the Adolescent Internet Adaptation Scale developed by Ji Aitong (9). This 17-item questionnaire consists of four dimensions: internet interpersonal orientation, academic evasion, problematic internet behavior, and rational internet use. “Internet Interpersonal Orientation” primarily refers to a heightened sense of comfort in online environments compared to real-world settings. Individuals with a stronger internet interpersonal orientation prefer to establish and maintain social connections online. “Academic Evasion” denotes the use of the internet as a tool for academic dishonesty or as a means to divert from educational tasks in unproductive ways. “Internet Problematic Behavior” characterizes online actions such as cyberbullying, attacking others, and other forms of disruptive behaviors. “Rational Internet Use” pertains to an individual’s prudent utilization of the internet, ensuring adherence to established online usage norms and guidelines. Owing to the fact that the dimensions of internet interpersonal orientation, academic evasion, and internet problematic behaviors evaluate maladaptive behaviors in internet usage, they can be categorized under the negative adaptation dimension. Conversely, internet rational use, which assesses behaviors from a constructive standpoint, signifies proactive adaptation and can thus be designated as the positive adaptation dimension. A 5-point scoring system was used, ranging from “strongly disagree” (1 point) to “strongly agree” (5 points). Negative dimension scores were reverse scored and added to the positive dimension to get the total score. The questionnaire’s reliability (Cronbach’s *α* coefficient) ranged from 0.64 to 0.80 for each dimension, and 0.83 overall. Confirmatory factor analysis showed item factor loadings all above 0.40, RMSEA = 0.06, NFI = 0.89, CFI = 0.92, GFI = 0.94, TLI = 0.90, indicating good reliability and validity (9).

#### 2.2.3 Internet cultural adaptation questionnaire

This study employed the standardized “Internet Cultural Adaptation Questionnaire” compiled by Huang Lifeng (18). This instrument was developed by referencing the “Adolescence Psychological Adaptation Scale (APAS)” constructed by Chen Huichang et al. (1995), the “Sociocultural Adaptation Scale” advanced by Ward & Kennedy (1999), the “Student Adaptation to College Questionnaire (SACQ)” by Gerdes & Mallinckrodt (1994), the “Chinese College Student Adaptation Scale” devised by the Ministry of Education’s “College Student Mental Health Assessment System” project team (2005), and the “University Student Internet Psychological Survey Questionnaire” formulated by Meng Ping (2003). Based on the fundamental understanding of internet cultural adaptation, 42 items were included in the questionnaire. It adopts a 5-point Likert scale: 1 – Strongly Disagree, 2 – Disagree, 3 – Neutral, 4 – Agree, 5 – Strongly Agree. Participants were instructed to select options based on their real-life experiences.

The questionnaire encompasses several domains, including Value Adaptation (assessing and adapting to the values of online culture), Social Adaptation (adapting to online activities conducive to internet society development), Cognitive Adaptation (adjusting to the basic understanding and operations of the internet), Psychological Field Adaptation (aligning with the mental environment of the online world), Moral Adaptation (evaluating ethical standards of online behaviors), Communication Adaptation (adapting to communication activities utilized in the internet realm for conveying thoughts, exchanging emotions, and coordinating interpersonal relationships), and Rule Adaptation (conforming to behavioral constraints set by online rules). The reliability of the questionnaire, as reflected by its Cronbach’s *α* coefficient, stands at 0.805. The KMO measure is 0.803, and the Bartlett Test significance level is 0.000, indicating significant differentiation. Consequently, the questionnaire boasts commendable validity and reliability (18).

### 2.3 Research process

In this study, a random sampling survey was conducted among adolescents in China based on the “sojum” of China Data Acquisition Company, and standardized questionnaires were used for testing. SPSS version 24.0 and AMOS version 24.0 were used for data analysis. The preliminary analysis was conducted by t test and reported the current state of adolescent internet addiction, internet adaptation, and internet cultural adaptation, and explored differences between these variables under different sociodemographic characteristics. The study tested the hypothesis model using structural equation modeling (SEM). In the model, negative internet adaptation, internet cultural adaptation, and internet addiction in adolescents were treated as latent variables, and their measurement indicators were classified using the isolated approach. The indirect effects of internet addiction were estimated using the bias-corrected bootstrapping approach (*N* = 1,000). Parameter estimates of the model were obtained using the maximum likelihood estimation procedure. The model fit was evaluated based on the following standards: GFI and AGFI ≥0.80, CFI, TLI, and RFI ≥0.90, and RMSEA ≤0.08.

## 3 Results

### 3.1 Differential analysis

The independent variables gender, place of origin, and grade were taken separately, with Internet addiction and its various dimensions, Internet adaptation and its various dimensions, and Internet cultural adaptation and its various dimensions as the dependent variables, to conduct independent sample *t*-tests.

The *t*-test results with gender as the independent variable showed (see Table 1) that on the levels of Internet addiction and its various dimensions, male Internet addiction scores were significantly higher than females in the aspect of interpersonal and health issues [*t*_(3119)_ = 3.448, *p <* 0.01, *d* = 0.12]; in terms of positive Internet adaptation, female scores for total Internet adaptation and reasonable Internet use were significantly higher than males [total Internet adaptation: *t*_(3119)_ = 2.144, *p <* 0.05, *d* = 0.08; reasonable Internet use: *t*_(3119)_ = 3.514, *p <* 0.001, *d* = 0.13]; in the dimension of negative Internet adaptation, female scores for academic escapism were significantly higher than males [*t*_(3119)_ = 3.513, *p <* 0.001, *d* = 0.13], and male scores for problematic Internet behavior were significantly higher than females [*t*_(3119)_ = 7.647, *p <* 0.001, *d* = 0.27]; on the levels of Internet cultural adaptation and its various dimensions, females scored significantly higher than males in terms of moral and rule adaptation to Internet culture [*t*_(3119)_ = 2.326, *p <* 0.05, *d* = 0.08; *t*_(3119)_ = 2.930, *p <* 0.01, *d* = 0.10].

**Table 1:** T-test for gender difference of all variables.

Variables	Male (*N* = 1,264) M (SD)	Female (*N* = 1857) M (SD)	*t*
Interpersonal and Health Problems	14.31(4.023)	13.82(3.730)	0.216**
Internet Adaptation (Total) Rational Internet Use Academic Evasion Internet Problematic Behavior Moral Adaptation Rule Adaptation	68.74(9.550) 15.23(3.560) 8.64(3.746) 4.62(2.077) 11.55(2.625) 11.41(2.514)	69.95(8.743) 15.67(3.627) 9.12(3.750) 4.10(1.680) 11.76(2.463) 11.67(2.313)	2.144** 3.514*** 3.513*** 7.647*** 2.326* 2.930**

Only results with significant differences are shown in the table.^**^Indicates a significant differences at the 0.01 level (two-tailed).^***^Indicates a significant differences at the 0.001 level (two-tailed).

The *t*-test results with place of origin as the independent variable showed (see Table 2) that at the levels of Internet addiction and its various dimensions, rural college students scored significantly higher than urban students [compulsive Internet use: *t*_(3119)_ = 5.027, *p* < 0.001, *d* = 0.18; withdrawal: *t*_(3119)_ = 4.551, *p* < 0.001, *d* = 0.16; tolerance: *t*_(3119)_ = 6.620, *p* < 0.001, *d* = 0.24; interpersonal and health issues: *t*_(3119)_ = 6.426, *p* < 0.001, *d* = 0.23; time management issues: *t*_(3119)_ = 7.180, *p* < 0.001, *d* = 0.26; total Internet addiction: *t*_(3119)_ = 6.628, *p* < 0.001, *d* = 0.24]; in terms of negative Internet adaptation, rural college students scored significantly higher than urban students [Internet interpersonal orientation: *t*_(3119)_ = 4.169, *p* < 0.001, *d* = 0.15; academic escapism: *t*_(3119)_ = 5.868, *p* < 0.001, *d* = 0.21; problematic Internet behavior: *t*_(3119)_ = 3.355, *p* < 0.01, *d* = 0.12], and rural college students scored significantly lower than urban students in terms of total Internet adaptation [*t*_(3119)_ = −5.206, *p* < 0.001, *d* = 0.19]; in terms of Internet cultural adaptation, rural college students scored significantly higher than urban students in psychological field adaptation and communication adaptation, but scored significantly lower in moral adaptation [psychological field adaptation: *t*_(3119)_ = 3.795, *p* < 0.001, *d* = 0.14; communication adaptation: *t*_(3119)_ = 3.448, *p* < 0.001, *d* = 0.14; moral adaptation: *t*_(3119)_ = −2.595, *p* < 0.01, *d* = 0.09].

**Table 2:** T-test with students’ place of birth difference of all variables.

Variables	Rural students (*N* = 1,128) M (SD)	Urban students (*N* = 1939) M (SD)	*t*
Compulsive Internet Use	10.63 (2.743)	10.08 (3.040)	5.027***
Internet Withdrawal Reaction Internet Addiction Tolerance Interpersonal and Health Problems Time Management Problems Internet Addiction (Total) Internet Interpersonal Orientation Academic Evasion Internet Problematic Behavior Internet Adaptation (Total) Psychological Field Adaptation Moral Adaptation Communication Adaptation	11.00 (2.672) 8.98 (2.116) 14.58 (3.558) 10.32 (2.616) 55.51 (12.086) 11.53 (4.564) 9.43 (3.671) 4.45 (1.892) 68.08 (8.882) 11.24 (2.903) 11.53 (2.445) 9.48 (2.482)	10.51 (3.032) 8.43 (2.322) 13.67 (3.992) 9.60 (2.769) 52.30 (13.699) 10.83 (4.494) 8.62 (3.774) 4.22 (1.848) 69.82 (9.145) 10.83 (2.952) 11.77 (2.579) 9.10 (2.560)	4.551** 6.620*** 6.426*** 7.180*** 6.628*** 4.169*** 5.868*** 3.355*** −5.206*** 3.795*** −2.595* 4.035***

Only results with significant differences are shown in the table.^**^Indicates a significant differences at the 0.01 level (two-tailed).^***^Indicates a significant differences at the 0.001 level (two-tailed).

The *t*-test results with grade as the independent variable showed (see Table 3) that at the levels of Internet addiction and its various dimensions, high school students scored significantly higher than middle school students [compulsive Internet use: *t*_(3119)_ = 3.763, *p* < 0.001, *d* = 0.13; withdrawal: *t*_(3119)_ = 3.471, *p* < 0.001, *d* = 0.12; tolerance: *t*_(3119)_ = 6.201, *p* < 0.001, *d* = 0.22; interpersonal and health issues: *t*_(3119)_ = 4.400, *p* < 0.001, *d* = 0.16; time management issues: *t*_(3119)_ = 7.135, *p* < 0.001, *d* = 0.26; total Internet addiction: *t*_(3119)_ = 5.429, *p* < 0.001, *d* = 0.19]; in terms of negative Internet adaptation, high school students scored significantly higher than middle school students [Internet interpersonal orientation: *t*_(3119)_ = 4.667, *p* < 0.001, *d* = 0.17; academic escapism: *t*_(3119)_ = 5.816, *p* < 0.001, *d* = 0.21; problematic Internet behavior: *t*_(3119)_ = 4.540, *p* < 0.001, *d* = 0.16], and middle school students scored significantly higher than high school students in total Internet adaptation [*t*_(3119)_ = 4.960, *p* < 0.01, *d* = 0.18]; at the levels of Internet cultural adaptation and its various dimensions, middle school students scored significantly lower than high school students in total Internet cultural adaptation, value adaptation, social adaptation, cognitive adaptation, psychological field adaptation, and communication adaptation [total Internet cultural adaptation: *t*_(3119)_ = −6.273, *p* < 0.001, *d* = 0.22; value adaptation: *t*_(3119)_ = −3.085, *p* < 0.01, *d* = 0.11; social adaptation: *t*_(3119)_ = −6.572, *p* < 0.001, *d* = 0.24; cognitive adaptation: *t*_(3119)_ = −4.626, *p* < 0.001, *d* = 0.17; psychological field adaptation: *t*_(3119)_ = −5.534, *p* < 0.001, *d* = 0.20; communication adaptation: *t*_(3119)_ = −6.273, *p* < 0.001, *d* = 0.22].

**Table 3:** T-test for grade difference of all variables.

Variables	Middle school students (*N* = 1,633) M (SD)	High school students (*N* = 1,488) M (SD)	*t*
Compulsive Internet Use	10.10 (3.094)	10.49 (2.753)	−3.763***
Internet Withdrawal Reaction Internet Addiction Tolerance Interpersonal and Health Problems Time Management Problems Internet Addiction (Total) Internet Interpersonal Orientation Academic Evasion Internet Problematic Behavior Internet Adaptation (Total) Value Adaptation Social Adaptation Cognitive Adaptation Psychological Field Adaptation Communication Adaptation Internet cultural adaptation (Total)	10.52 (3.064) 8.40 (2.321) 13.73 (4.030) 9.54 (2.798) 52.30 (13.854) 10.73 (4.449) 8.56 (3.763) 4.16 (1.787) 69.93 (9.120) 13.34 (3.224) 15.33 (4.665) 18.62 (3.954) 10.70 (2.882) 8.82 (2.603) 89.99 (16.656)	10.88 (2.719) 8.90 (2.166) 14.33 (3.635) 10.24 (2.614) 54.85 (12.312) 11.49 (4.592) 9.33 (3.705) 4.47 (1.941) 68.32 (8.972) 13.69 (3.069) 16.40 (4.314) 19.24 (3.579) 11.29 (2.973) 9.72 (2.375) 93.64 (15.695)	−3.471** −6.201*** −4.400*** −7.135*** −5.429*** −4.667*** −5.816*** −4.540*** 4.960*** −3.085*** −6.572*** −6.426*** −5.534*** −10.066*** −6.273***

Only results with significant differences are shown in the table.^**^Indicates a significant differences at the 0.01 level (two-tailed).^***^Indicates a significant differences at the 0.001 level (two-tailed).

### 3.2 Correlation analysis

Internet adaptation’s online interpersonal orientation and academic avoidance are significantly positively correlated with the overall score of Internet cultural adaptation as well as its sub-dimensions: value adaptation, social adaptation, cognitive adaptation, psychological field adaptation, and communication adaptation (*p*s < 0.001). Internet problematic behaviors show a significant positive correlation with the value adaptation, social adaptation, psychological field adaptation, communication adaptation, and the overall score of Internet cultural adaptation (*p*s < 0.001). However, they are significantly negatively correlated with rule adaptation and moral adaptation (*p*s < 0.001). Rational use of the Internet under the domain of Internet adaptation has a significant positive correlation with the overall score of Internet cultural adaptation and all its sub-dimensions, excluding psychological field adaptation (*p*s < 0.001).

The overall score of Internet addiction and its various dimensions have a significant positive correlation with the negative adaptation dimensions of the Internet (*p*s < 0.001) and a significant negative correlation with rational Internet use and the overall score of Internet adaptation (*p*s < 0.001).

Value adaptation and social adaptation under Internet cultural adaptation are significantly positively correlated with compulsive Internet use, withdrawal reactions, tolerance, time management issues, and the overall score of Internet addiction (*p*s < 0.01). Cognitive adaptation shows a significant negative correlation with interpersonal and health issues related to Internet addiction, time management issues, and the overall score of Internet addiction (*p*s < 0.001). Psychological field adaptation and communication adaptation have a significant positive correlation with the overall score and all dimensions of Internet addiction (*p*s < 0.001). Moral adaptation and rule adaptation are significantly negatively correlated with the overall score and all dimensions of Internet addiction (*p* < 0.001). The overall score of Internet cultural adaptation is significantly positively correlated with compulsive Internet use, withdrawal reactions, tolerance, time management, and the overall score of Internet addiction (*p*s < 0.01) (see Table 4).

**Table 4:** Correlation analysis among variables.

	Compulsive Internet Use	Internet Withdrawal Reaction	Internet Addiction Tolerance	Interpersonal and Health Problems	Time Management Problems	Internet Addiction (total)	Internet Interpersonal Orientation	Academic Evasion	Internet Problematic Behavior	Rational Internet Use	Internet Adaptation (total)	Value Adaptation	Social Adaptation	Cognitive Adaptation	Psychological Field Adaptation	Moral Adaptation	Communication Adaptation	Rule Adaptation	Internet Culture Adaptation (total)
Compulsive Internet Use	1	0.815^**^	0.809^**^	0.809^**^	0.734^**^	0.929^**^	−0.471^**^	−0.632^**^	−0.281^**^	−0.117^**^	−0.599^**^	0.100^**^	0.054^**^	−0.034	0.155^**^	−0.085^**^	0.169^**^	−0.057^**^	0.059^**^
Internet Withdrawal Reaction	0.815^**^	1	0.798^**^	0.702^**^	0.668^**^	0.882^**^	−0.448^**^	−0.565^**^	−0.270^**^	−0.121^**^	−0.559^**^	0.128^**^	0.070^**^	−0.014	0.140^**^	−0.070^**^	0.177^**^	−0.046^**^	0.076^**^
Internet Addiction Tolerance	0.809^**^	0.798^**^	1	0.741^**^	0.719^**^	0.893^**^	−0.437^**^	−0.595^**^	−0.277^**^	−0.118^**^	−0.566^**^	0.093^**^	0.055^**^	−0.026	0.147^**^	−0.071^**^	0.166^**^	−0.049^**^	0.061^**^
Interpersonal and Health Problems	0.809^**^	0.702^**^	0.741^**^	1	0.763^**^	0.912^**^	−0.453^**^	−0.559^**^	−0.296^**^	−0.172^**^	−0.584^**^	0.025	0.007	−0.103^**^	0.176^**^	−0.147^**^	0.126^**^	−0.112^**^	−0.005
Time Management Problems	0.734^**^	0.668^**^	0.719^**^	0.763^**^	1	0.864^**^	−0.439^**^	−0.546^**^	−0.322^**^	−0.156^**^	−0.571^**^	0.077^**^	0.073^**^	−0.040^*^	0.154^**^	−0.111^**^	0.157^**^	−0.090^**^	0.047^**^
Internet Addiction (total)	0.929^**^	0.882^**^	0.893^**^	0.912^**^	0.864^**^	1	−0.502^**^	−0.644^**^	−0.323^**^	−0.156^**^	−0.643^**^	0.089^**^	0.054^**^	−0.054^**^	0.174^**^	−0.112^**^	0.174^**^	−0.083^**^	0.049^**^
Internet Interpersonal Orientation	−0.471^**^	−0.448^**^	−0.437^**^	−0.453^**^	−0.439^**^	−0.502^**^	1	0.524^**^	0.455^**^	−0.040^*^	0.794^**^	−0.282^**^	−0.316^**^	−0.077^**^	−0.253^**^	−0.016	−0.293^**^	−0.020	−0.256^**^
Academic Evasion	−0.632^**^	−0.565^**^	−0.595^**^	−0.559^**^	−0.546^**^	−0.644^**^	0.524^**^	1	0.338^**^	0.050^**^	0.764^**^	−0.148^**^	−0.075^**^	−0.026	−0.163^**^	0.014	−0.215^**^	−0.010	−0.118^**^
Internet Problematic Behavior	−0.281^**^	−0.270^**^	−0.277^**^	−0.296^**^	−0.322^**^	−0.323^**^	0.455^**^	0.338^**^	1	0.146^**^	0.628^**^	−0.067^**^	−0.154^**^	0.042^*^	−0.211^**^	0.121^**^	−0.175^**^	0.128^**^	−0.073^**^
Rational Internet Use	−0.117^**^	−0.121^**^	−0.118^**^	−0.172^**^	−0.156^**^	−0.156^**^	−0.040^*^	0.050^**^	0.146^**^	1	0.414^**^	0.237^**^	0.193^**^	0.330^**^	0.012	0.364^**^	0.156^**^	0.359^**^	0.312^**^
Internet Adaptation (total)	−0.599^**^	−0.559^**^	−0.566^**^	−0.584^**^	−0.571^**^	−0.643^**^	0.794^**^	0.764^**^	0.628^**^	0.414^**^	1	−0.125^**^	−0.146^**^	0.086^**^	−0.232^**^	0.163^**^	−0.211^**^	0.150^**^	−0.072^**^
Value Adaptation	0.100^**^	0.128^**^	0.093^**^	0.025	0.077^**^	0.089^**^	−0.282^**^	−0.148^**^	−0.067^**^	0.237^**^	−0.125^**^	1	0.614^**^	0.558^**^	0.350^**^	0.582^**^	0.651^**^	0.529^**^	0.826^**^
Social Adaptation	0.054^**^	0.070^**^	0.055^**^	0.007	0.073^**^	0.054^**^	−0.316^**^	−0.075^**^	−0.154^**^	0.193^**^	−0.146^**^	0.614^**^	1	0.551^**^	0.433^**^	0.423^**^	0.656^**^	0.373^**^	0.825^**^
Cognitive Adaptation	−0.034	−0.014	−0.026	−0.103^**^	−0.040^*^	−0.054^**^	−0.077^**^	−0.026	0.042^*^	0.330^**^	0.086^**^	0.558^**^	0.551^**^	1	0.192^**^	0.634^**^	0.491^**^	0.589^**^	0.789^**^
Psychological Field Adaptation	0.155^**^	0.140^**^	0.147^**^	0.176^**^	0.154^**^	0.174^**^	−0.253^**^	−0.163^**^	−0.211^**^	0.012	−0.232^**^	0.350^**^	0.433^**^	0.192^**^	1	0.149^**^	0.521^**^	0.154^**^	0.540^**^
Moral Adaptation	−0.085^**^	−0.070^**^	−0.071^**^	−0.147^**^	−0.111^**^	−0.112^**^	−0.016	0.014	0.121^**^	0.364^**^	0.163^**^	0.582^**^	0.423^**^	0.634^**^	0.149^**^	1	0.349^**^	0.666^**^	0.712^**^
Communication Adaptation	0.169^**^	0.177^**^	0.166^**^	0.126^**^	0.157^**^	0.174^**^	−0.293^**^	−0.215^**^	−0.175^**^	0.156^**^	−0.211^**^	0.651^**^	0.656^**^	0.491^**^	0.521^**^	0.349^**^	1	0.345^**^	0.777^**^
Rule Adaptation	−0.057^**^	−0.046^**^	−0.049^**^	−0.112^**^	−0.090^**^	−0.083^**^	−0.020	−0.010	0.128^**^	0.359^**^	0.150^**^	0.529^**^	0.373^**^	0.589^**^	0.154^**^	0.666^**^	0.345^**^	1	0.675^**^
Internet Culture Adaptation (total)	0.059^**^	0.076^**^	0.061^**^	−0.005	0.047^**^	0.049^**^	−0.256^**^	−0.118^**^	−0.073^**^	0.312^**^	−0.072^**^	0.826^**^	0.825^**^	0.789^**^	0.540^**^	0.712^**^	0.777^**^	0.675^**^	1

^*^Indicates a significant correlation at the 0.05 level (two-tailed), ^**^indicates a significant correlation at the 0.01 level (two-tailed), and ^***^indicates a significant correlation at the 0.001 level (two-tailed).

### 3.3 Measurement model and hypothetical model

**Figure 1:**
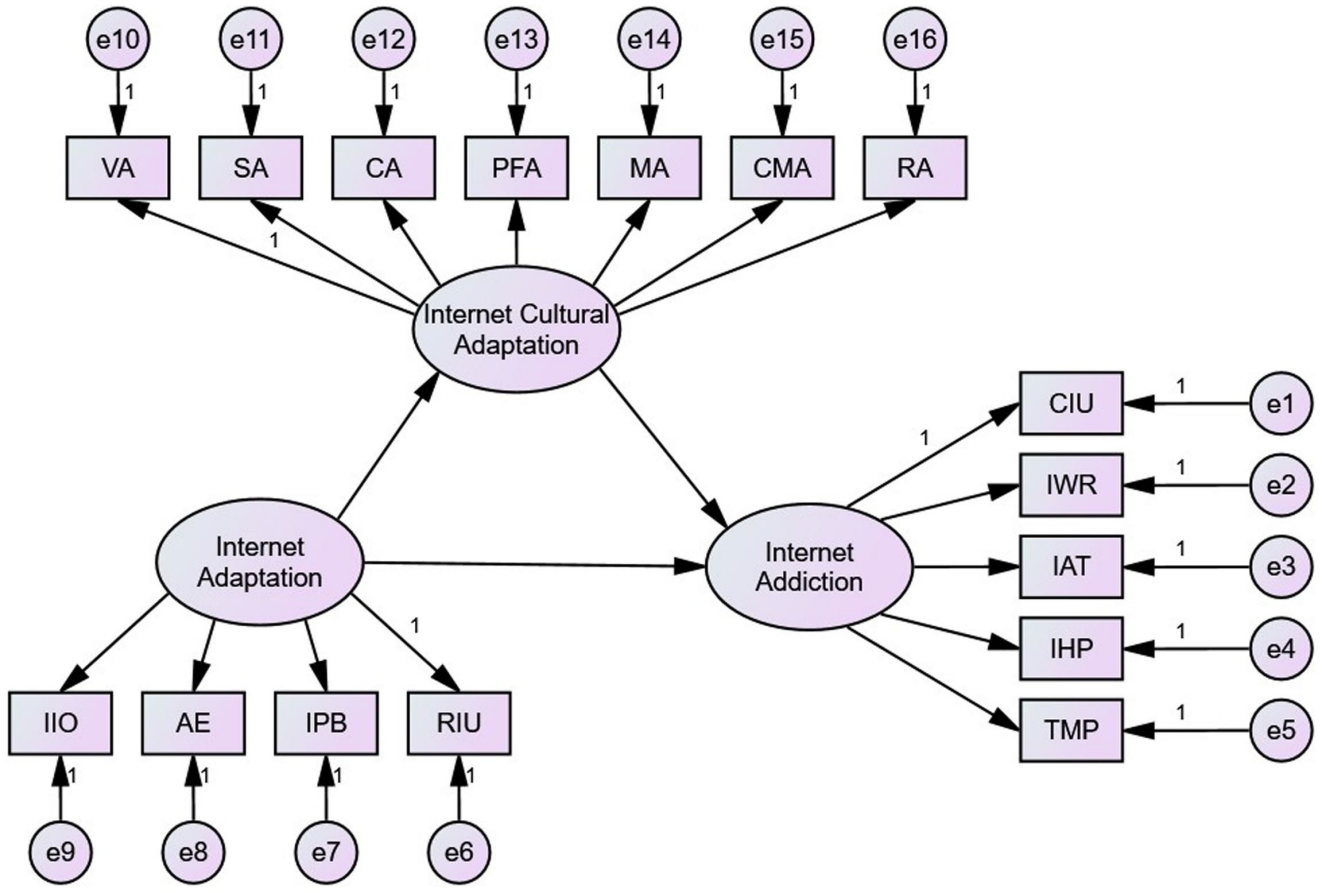
Hypothesis model of the relationship among internet adaptation, internet addiction, and internet cultural adaptation. CIU stands for Compulsive Internet Use; IWR stands for internet withdrawal reaction interpersonal and health problems; IAT stands for internet addiction tolerance; IHP stands for interpersonal and health problems; TMP stands for time management problems; IIO stands for Internet Interpersonal Orientation; AE stands for Academic Evasion; IPB stands for Internet Problematic Behavior; RIU stands for Rational Internet Use; VA stands for Value Adaptation; SA stands for Social Adaptation; CA stands for Cognitive Adaptation; PFA stands for Psychological Field Adaptation; MA stands for Moral Adaptation; CMA stands for Communication Adaptation; RA stands for Rule Adaptation.

This study hypothesizes that “Negative Internet Adaptation” can directly lead to Internet addiction, and this relationship may also be influenced by “Internet Cultural Adaptation” (see Figure 1).

Confirmatory factor analysis was performed on the dimensions of Internet addiction, Internet adaptation, and Internet cultural adaptation. Results are presented in Table 5. The factor loadings for “Rational Internet Use” and “Psychological Field Adaptation” were 0.065 and 0.187 respectively, and thus, they were excluded. The factor loadings for the remaining dimensions were all greater than 0.36, suggesting an acceptable overall fit of the model.

**Table 5:** Parameter estimation results of structural equation model.

			B	S.E.	C.R.	*p*	*β*	SMC (R2)
Internet Cultural Adaptation	←	Internet Adaptation	−0.67	0.07	−9.537	***	−0.224	−0.67
Internet Addiction	←	Internet Cultural adaptation	−0.132	0.017	−7.811	***	−0.126	−0.132
Internet Addiction	←	Internet Adaptation	−2.605	0.112	−23.267	***	−0.829	−2.517
Compulsive Internet Use	←	Internet Addiction	1				0.927	0.927
Internet Withdrawal Reaction	←	Internet Addiction	0.921	0.012	74.874	***	0.864	0.864
Internet Addiction Tolerance	←	Internet Addiction	0.731	0.009	79.016	***	0.882	0.882
Interpersonal and Health Problems	←	Internet Addiction	1.219	0.016	74.502	***	0.862	0.862
Time Management Problems	←	Internet Addiction	0.814	0.013	65.061	***	0.813	0.813
Internet Problematic Behavior	←	Internet Adaptation	1				0.465	0.465
Rational Internet Use	←	Internet Adaptation	0.261	0.08	3.268	***	0.065	0.065
Academic Evasion	←	Internet Adaptation	3.434	0.144	23.783	***	0.794	0.794
Internet Interpersonal Orientation	←	Internet Adaptation	3.594	0.158	22.757	***	0.688	0.688
Value Adaptation	←	Internet Cultural Adaptation	1				0.828	0.828
Social Adaptation	←	Internet Cultural Adaptation	1.303	0.029	45.101	***	0.748	0.748
Cognitive Adaptation	←	Internet Cultural Adaptation	1.071	0.024	44.102	***	0.735	0.735
Moral Adaptation	←	Internet Cultural Adaptation	0.664	0.017	40.223	***	0.683	0.683
Communication Adaptation	←	Internet Cultural Adaptation	0.719	0.016	44.264	***	0.737	0.737
Rule Adaptation	←	Internet Cultural Adaptation	0.591	0.016	37.22	***	0.641	0.641
Psychological Field Adaptation	←	Internet Cultural Adaptation	0.488	0.021	23.709	***	0.432	0.432

^***^Indicates a significant correlation at the 0.001 level (two-tailed).

In the hypothetical model, “Online Interpersonal Orientation,” “Academic Avoidance,” and “Internet Problematic Behaviors” are all reverse-scored to consistently reflect the level of positive Internet adaptation in line with “Rational Internet Use.” Therefore, “Negative Internet Adaptation” can be set as a latent variable, with the original values of “Online Interpersonal Orientation,” “Academic Avoidance,” and “Internet Problematic Behaviors” serving as its observed variables.

### 3.4 Structural equation model

Upon using the Maximum Likelihood Estimates (MLE) for the continuity fitting of the initial model (detailed in Table 6), based on Modification Indices (M.I.) and theoretical rationale, not only was there significant covariation between the two factors “Rule Adaptation” and “Moral Adaptation” within “Internet Cultural Adaptation” (M.I_Moral-Rule_ = 497.089, *r* = 0.666, *p* < 0.001), but they also exhibited substantial covariation with other factors (e.g., M.I_Moral-Communication_ = 367.663, *r* = 0.349, *p* < 0.001).

**Table 6:** Parameter estimation results of modified structural equation model.

			B	S.E.	C.R.	*p*	*β*	SMC (R2)
Internet Cultural Adaptation	←	Negative Internet Adaptation	0.893	0.071	12.543	***	0.313	0.313
Internet Addiction	←	Negative Internet Adaptation	−0.16	0.019	−8.484	***	−0.147	−0.147
Internet Addiction	←	Negative Internet Adaptation	2.614	0.112	23.301	***	0.843	0.796
Compulsive Internet Use	←	Internet Addiction	1				0.927	0.86
Internet Withdrawal Reaction	←	Internet Addiction	0.921	0.012	74.896	***	0.864	0.746
Internet Addiction Tolerance	←	Internet Addiction	0.731	0.009	79.017	***	0.882	0.778
Interpersonal and Health Problems	←	Internet Addiction	1.219	0.016	74.474	***	0.862	0.743
Time Management Problems	←	Internet Addiction	0.814	0.013	65.033	***	0.813	0.66
Internet Problematic Behavior	←	Negative Internet Adaptation	1				0.471	0.222
Academic Evasion	←	Negative Internet Adaptation	3.338	0.139	24.05	***	0.782	0.611
Internet Interpersonal Orientation	←	Negative Internet Adaptation	3.615	0.156	23.222	***	0.701	0.492
Value Adaptation	←	Internet cultural adaptation	1				0.798	0.637
Social Adaptation	←	Internet cultural adaptation	1.445	0.032	44.861	***	0.799	0.638
Cognitive Adaptation	←	Internet Cultural Adaptation	0.994	0.027	36.332	***	0.657	0.432
Communication Adaptation	←	Internet Cultural Adaptation	0.819	0.018	45.374	***	0.81	0.656

**Figure 2:**
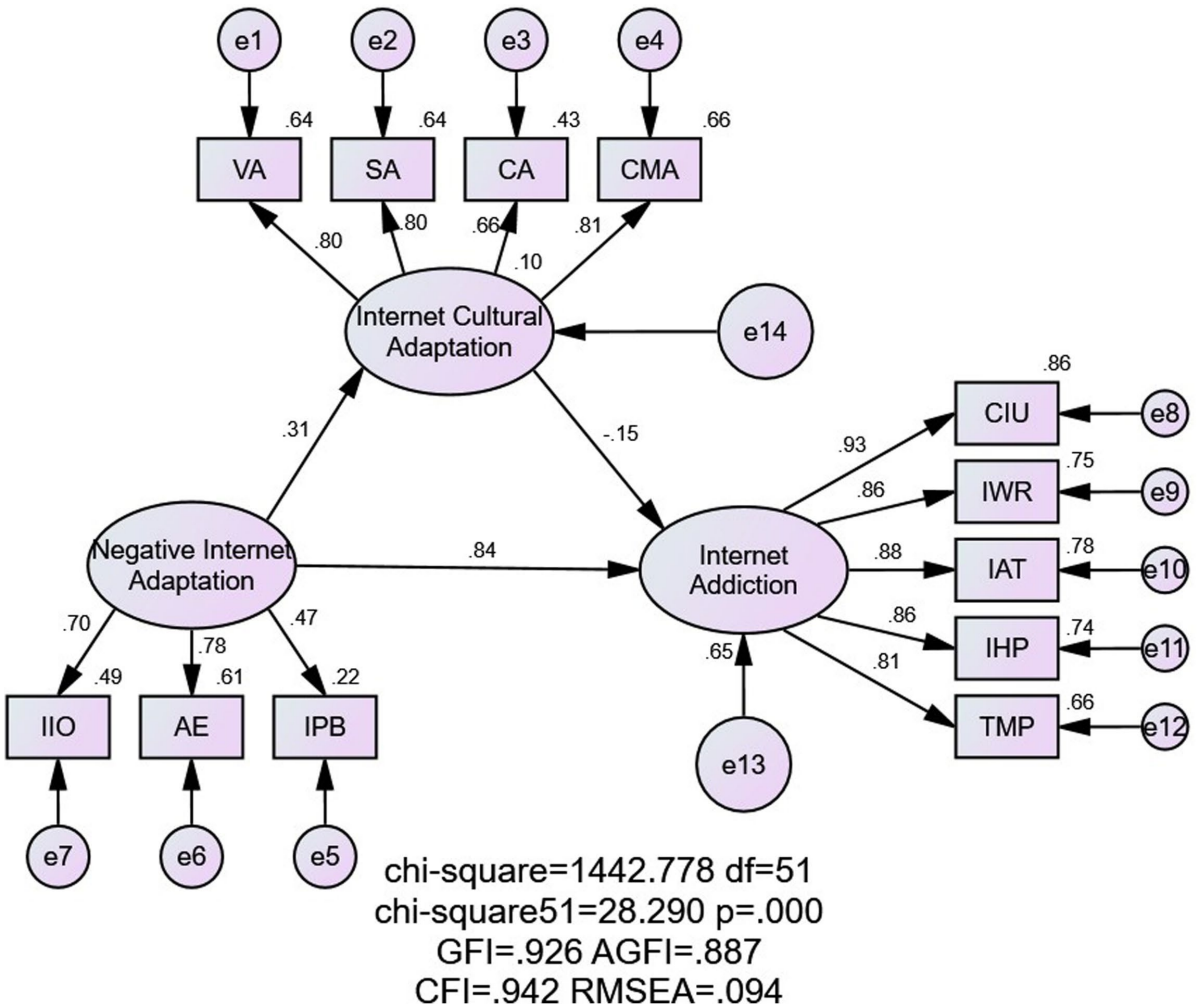
The SEM of the relationship among internet adaptation, internet addiction and internet cultural adaptation. SEM stands for structural equation model; CIU stands for Compulsive Internet Use; IWR stands for internet withdrawal reaction interpersonal and health problems; IAT stands for internet addiction tolerance; IHP stands for interpersonal and health problems; TMP stands for time management problems; IIO stands for Internet Interpersonal Orientation; AE stands for Academic Evasion; IPB stands for Internet Problematic Behavior.

Taking into account the path coefficients of the initial model and the modification indices, the “Rule Adaptation” and “Moral Adaptation” factors were removed from the measurement of Internet cultural adaptation to improve the model’s fit and explanatory power. Existing research indicates that when the sample size is relatively large (*N* > 1,000), the chi-square test may not serve as a crucial model fit index. Also, based on other fit indices, an RMSEA threshold between 0.08 and 0.10 is acceptable (19, 20). Overall, the model demonstrated a good fit [*χ*2 = 1442.778 (*df* = 51), *p* < 0.001, GFI = 0.926, CFI = 0.942, AGFI = 0.887, RMSEA (90% CI) = 0.094 [0.089, 0.098], RFI = 0.922, TLI = 0.925]. The fit indices and their respective evaluation criteria are shown in Table 7. The final structural equation model is shown in Figure 2.

**Table 7:** Model fitting indexes and fit criteria.

Indices	*χ*^2^/df	*p*	GFI	CFI	AGFI	RMSEA	RFI	TLI
Fit criteria	1–5	>0.05	>0.8	>0.9	>0.8	<0.08	>0.90	>0.90
The model	28.290	<0.001	0.926	0.942	0.887	0.094	0.922	0.925

All factor loadings had *p*-values less than 0.001, indicating that the three latent variables (Internet Adaptation, Internet Cultural Adaptation, and Internet Addiction) meaningfully explained the measurement variables (scale data). Moreover, the path coefficients among these latent variables were significant.

Negative Internet Adaptation had a direct positive effect on Internet Addiction (estimates: 0.843, SE = 0.112, *p* < 0.001), while Internet Cultural Adaptation had a direct negative effect on Internet Addiction (estimates: −0.147, SE = 0.019, *p* < 0.001). Negative Internet Adaptation also had a direct positive effect on Internet Cultural Adaptation (estimates: 0.313, SE = 0.071, *p* < 0.001). The direct effect of Negative Internet Adaptation on Internet Addiction was 0.843, the indirect effect was −0.046, and the total effect was 0.796. Notably, the signs of the direct and indirect effects were opposite, with the absolute value of the total effect being lower than expected, indicating a masking of the total effect. A bias-corrected percentile Bootstrap method (with 1,000 resamples) was used to test the mediation effect. Observing the bias-corrected 95% confidence interval’s upper and lower limits, the indirect effect of Internet Adaptation on Internet Addiction, mediated through Internet Cultural Adaptation, was significant (estimates: −0.046, SE = 0.071, *p* < 0.001), accounting for 6% of the total effect.

## 4 Discussion

### 4.1 The influence of gender, grade level, and place of origin on adolescents’ internet adaptation, internet cultural adaptation, and internet addiction

Internet adaptation refers to an individual’s ability to adapt to the internet environment, which is crucial for modern living and working (21). This study found that gender, place of origin, and grade level significantly affect internet adaptation. Regarding gender, consistent research results show that males have better internet adaptation ability than females (22, 23), possibly because males exhibit higher technology usage frequency and self-confidence in technology. From the place of origin perspective, students born in urban areas have an advantage in internet adaptation ability compared to those born in rural areas (24, 25), likely due to the relatively abundant internet facilities and access resources in urban areas. Regarding grade level, higher-grade students tend to have stronger internet adaptation abilities and more positive attitudes towards the internet, possibly because they view it more as a teaching and practical tool and believe it offers greater assistance to their studies and research. Hence, they accumulate more internet usage experience in their studies and daily life (26–28).

Internet cultural adaptation refers to an individual’s adaptation level within internet culture. This study found that gender, place of origin, and grade level all significantly impact internet cultural adaptation. Males generally perform better in internet cultural adaptation, possibly because they are more willing than females to accept and adapt to new ideas and behavior patterns in the internet culture (29–32). The place of origin affects students’ internet cultural adaptation ability, where urban students adapt better to internet culture than rural students, possibly because urban youths have easier access to internet resources and technology and face more opportunities to use the internet (33, 34).

The adaptation of high school students to internet culture can be attributed to several factors, especially when compared to middle school students. Firstly, as adolescents grow older, they generally develop more sophisticated cognitive and emotional skills, which help them navigate the complexities of the digital world with greater ease and maturity. This development is evident in the way older teens, typically high school students, engage with social media and technology.

Internet addiction refers to the phenomenon where individuals cannot control their excessive use of the internet, considered a possible psychological barrier (35). This study found that gender, place of origin, and grade level are all significant influencing factors of internet addiction. Gender impacts the degree of internet addiction, with research finding that males are more likely to be addicted to the internet than females. This may be because males use the internet more frequently and for longer durations than females (36). Regarding place of origin, students born in Chinese cities have a higher probability of internet addiction than those born in rural areas, possibly because urban students are exposed to the internet and richer internet content earlier, leading to dependency (37). However, some recent studies indicate that rural youths or university students have higher rates of positive internet addiction tests, short video dependency, and mobile phone addiction than those in cities (38, 9). As grade level increases, the phenomenon of internet addiction also increases, possibly because higher-grade students spend more time on the internet, enhancing its attractiveness and dependency (39).

### 4.2 Relationship between negative internet adaptation and internet addiction

This study found that the total score of internet addiction and its dimensions were significantly positively correlated with the dimensions of negative internet adaptation. Negative internet adaptation is a concept that covers many aspects; it mainly describes the poor adaptation cognition of adolescents in the internet environment and the resulting over-reliance on and negative emotional reactions to the internet. This adaptation difficulty may stem from the complexity, uncertainty, and potential negative interpersonal interactions (such as cyberbullying) of the internet environment. These factors may make adolescents feel confused and pressured, leading them to adopt negative coping strategies, such as excessive internet use to escape reality, thereby increasing the risk of internet addiction (40, 41).

Firstly, negative internet adaptation may trigger a series of negative emotional reactions, such as anxiety, aversion, depression, and helplessness (42–44). These negative emotions may further exacerbate adolescents’ over-reliance on the internet because the internet can serve as a temporary emotional regulation tool to help them escape these unpleasant feelings (45). However, this method of emotional regulation is short-lived and fragile because it does not truly address the adolescents’ adaptation problems but may instead increase their risk of internet addiction (46).

Secondly, from the perspective of interpersonal interaction theory, negative internet adaptation may hinder adolescents from establishing healthy and satisfying interpersonal relationships (47, 48). The complexity and uncertainty of the internet environment may make adolescents feel troubled and frustrated, causing them to experience disappointment and frustration in interpersonal interactions. This situation may make them more inclined to cope negatively, such as by excessively using the internet to avoid the troubles of interpersonal relationships, thereby increasing the risk of internet addiction (49).

### 4.3 Relationship between internet adaptation and internet cultural adaptation

In this study, it is found that the network problem behaviors of negative network adaptation are negatively correlated with rule adaptation and moral adaptation, that is, the more problem behaviors such as cyberbullying, the worse the college students’ ability to adapt to network rules and distinguish right from good from evil in the network field. These findings are reasonable. However, it is worth discussing that the study also reveals that the dimensions of negative Internet adaptation (Internet problem behavior, academic avoidance and Internet problem behavior) are significantly positively correlated with the most multiple dimensions of Internet acculturation and their total scores. In other words, the more negative the tendency of Internet interpersonal relationship, the more obvious the tendency of Internet evasion, or the more Internet problem behavior of college students. Then they may be more culturally adapted to the Internet. In general, there is a positive correlation between negative internet adaptation and internet cultural adaptation, that is, the higher the degree of negative internet adaptation, the higher the degree of internet cultural adaptation. This relationship is somewhat consistent with common sense and can be explained by various theories. According to the “stress adaptation model” (50, 51), when faced with stress and challenges in the internet environment (i.e., negative internet adaptation), adolescents may try to alleviate stress by adapting more deeply to internet culture (i.e., internet cultural adaptation). In other words, negative internet adaptation may prompt adolescents to immerse themselves more in internet culture to obtain resources and information they believe can help solve their problems, leading to a positive correlation between negative internet adaptation and internet cultural adaptation. The social comparison theory suggests that individuals tend to compare themselves with others, seeking social recognition and acceptance. In the internet environment, individuals with a high degree of negative internet adaptation may receive more attention and participation from other internet users, thus increasing their sense of presence and adaptation in internet culture (52, 53). Additionally, the social needs theory points out that individuals have a basic social need, that is, to establish connections, gain support, and communicate with others. In internet culture, individuals with a high degree of negative internet adaptation may rely more on internet social interactions and internet interactions to meet their social needs, thereby increasing their degree of adaptation to internet culture (54).

### 4.4 Role of internet cultural adaptation in predicting internet addiction from negative internet adaptation

This study shows that the impact of negative internet adaptation on internet addiction has both direct and indirect effects, and the signs of the two are opposite. The direct effect refers to the direct impact of negative internet adaptation on internet addiction, while the indirect effect refers to the way in which the relationship between negative internet adaptation and internet addiction is affected by mediating variables. The direct effect of negative internet adaptation on internet addiction is generally considered to be positive, that is, the higher the degree of negative internet adaptation, the more likely the individual is to fall into the risk of internet addiction (55, 56).

However, when introducing internet cultural adaptation as a mediating variable, the impact of negative internet adaptation on internet addiction may change. Internet cultural adaptation is believed to possibly play a mediating role between negative internet adaptation and internet addiction. Specifically, internet cultural adaptation may change the individual’s attitude and emotional response to the internet environment, thereby affecting its perception and handling of negative internet experiences. This study found that internet cultural adaptation played a negative moderating role between negative internet adaptation and internet addiction, that is, when the level of internet cultural adaptation is higher, the impact of negative internet adaptation on internet addiction becomes a negative relationship. This result suggests that improving the degree of internet cultural adaptation may help to alleviate the adverse effects of negative internet adaptation on internet addiction. Higher internet cultural adaptation may make individuals more capable of dealing with internet troubles and negative experiences, reducing the degree of negative internet adaptation. At the same time, internet cultural adaptation may also reduce the demand for internet addictive behaviors by providing positive internet participation and satisfaction, thereby reducing the risk of internet addiction (57–60).

## 5 Conclusion

Overall, the impact of negative internet adaptation on internet addiction is modulated by internet cultural adaptation. Internet cultural adaptation, by altering an individual’s attitudes and emotional responses towards the internet environment, as well as providing positive internet involvement and psychological need satisfaction, may mitigate the adverse effects of negative internet adaptation on internet addiction (61–63). This finding provides a crucial theoretical basis for a deeper understanding of the mechanisms behind the development of internet addiction, and also expands the theory of cross-cultural adaptation from the perspective of network acculturation, and the most outstanding contribution is to confirm that network acculturation maladjustment will lead to negative behavioral consequences as well as real acculturation difficulties. At the same time, this study offers new directions and strategies for its intervention and prevention, for example, education courses can be set to improve teenagers’ ability to adapt to internet culture, so as to reduce their negative internet adaptive behavior and internet addiction tendency.

## 6 Limitations

The target population of this study is focused on 13–17 years old Chinese adolescents, therefore, there is a certain limitation in the sample selection, which may affect the generalizability of the research results. In the future, it is necessary to include adolescents of all ages and different cultural backgrounds in the study. Additionally, this study mainly explores the association between internet cultural adaptation and internet addiction through questionnaire surveys. Although it can reveal the correlation between the two, it cannot definitively deduce a causal relationship. This methodological limitation may hinder our in-depth understanding of the problem. Furthermore, when exploring the influencing factors of adolescent internet addiction, this study did not fully consider other possible influencing factors, such as personality traits, parenting styles, and mood states. This may limit the complexity and effectiveness of the theoretical model constructed. Future research can explore these factors more comprehensively, extending the research content from Internet addiction to positive Internet behaviors (such as learning support, information acquisition and social interaction) to enrich and refine our understanding of the influence of adolescents’ Internet acculturation on their Internet behaviors.

## Data availability statement

The raw data supporting the conclusions of this article will be made available by the authors, without undue reservation.

## Ethics statement

The studies involving humans were approved by the Institutional Review Board of the Department of Psychology, Fujian Normal University and Fujian Agriculture and Forestry University. The studies were conducted in accordance with the local legislation and institutional requirements. Written informed consent for participation in this study was provided by the participants’ legal guardians/next of kin.

## Author contributions

YY: Conceptualization, Data curation, Formal analysis, Investigation, Methodology, Software, Validation, Writing – original draft, Writing – review & editing. JZ: Formal analysis, Funding acquisition, Investigation, Methodology, Writing – original draft, Writing – review & editing. YN: Data curation, Investigation, Methodology, Writing – original draft. YaF: Methodology, Writing – review & editing. YZ: Investigation, Writing – review & editing. YiF: Data curation, Writing – review & editing.

## References

[1] Young KS. Internet addiction: the emergence of a new clinical disorder. Cyberpsychol Behav. (2009) 1:237–44. doi: 10.1089/cpb.1998.1.237

[2] Young KS Abreu CN de Kecanduan Internet, Young K. S. Abreu C. N. de (Eds.). Pustaka Belajar. (2017).

[3] Young KS Rogers RC. The relationship between depression and internet addiction. Cyber Psychol Behav. (1998) 1:25–8. doi: 10.1089/cpb.1998.1.25

[4] Zhao Q Huang Y Li C. Does adolescents’ internet addiction trigger depressive symptoms and aggressive behavior, or vice versa? The moderating roles of peer relationships and gender. Comput Hum Behav. (2022) 129:107143. doi: 10.1016/j.chb.2021.107143

[5] Lin M-P. Prevalence of internet addiction during the COVID-19 outbreak and its risk factors among junior high school students in Taiwan. Int J Environ Res Public Health. (2020) 17:8547. doi: 10.3390/ijerph17228547, PMID: 33218018 PMC7698622

[6] Ozturk FO Ayaz-Alkaya S. Internet addiction and psychosocial problems among adolescents during the COVID-19 pandemic: a cross-sectional study. Arch Psychiatr Nurs. (2021) 35:595–601. doi: 10.1016/j.apnu.2021.08.007, PMID: 34861951 PMC8424060

[7] Sun Y Li Y Bao Y Meng S Sun Y Schumann G. Brief report: increased addictive internet and substance use behavior during the COVID-19 pandemic in China. Am J Addict. (2020) 29:268–70. doi: 10.1111/ajad.13066, PMID: 32500608 PMC7300868

[8] Jin CC Wang BC Ji AT. The relationship between the dark triad and internet adaptation among adolescents in China: internet use preference as a mediator. Front Psychol. (2019) 10:2023. doi: 10.3389/fpsyg.2019.02023, PMID: 31543856 PMC6729730

[9] Zhou Y Huang J Huang J Li J. Research on the status quo of college students’ short video dependence. Adv Psychol. (2022) 12:4136. doi: 10.12677/AP.2022.1212500

[10] Bi X Jin J. Psychological capital, college adaptation, and internet addiction: an analysis based on moderated mediation model. Front Psych. (2021) 12:712964. doi: 10.3389/fpsyt.2021.712964, PMID: 34899409 PMC8652336

[11] Lebni JY Toghroli R Abbas J NeJhaddadgar N Salahshoor MR Mansourian M. A study of internet addiction and its effects on mental health: a study based on Iranian university students. J Educ Health Promotion. (2020) 9:205. doi: 10.4103/jehp.jehp_148_20, PMID: 33062738 PMC7530416

[12] Kim YY. Cross-cultural adaptation. Oxford research encyclopedia of communication. (2017)

[13] Ward C Geeraert N. Advancing acculturation theory and research: the acculturation process in its ecological context. Curr Opin Psychol. (2016) 8:98–104. doi: 10.1016/j.copsyc.2015.09.021, PMID: 29506811

[14] Baloğlu M Şahin R Arpaci I. A review of recent research in problematic internet use: gender and cultural differences. Curr Opin Psychol. (2020) 36:124–9. doi: 10.1016/j.copsyc.2020.05.008, PMID: 32629412

[15] Borca G Bina M Keller PS Gilbert LR Begotti T. Internet use and developmental tasks: adolescents’ point of view. Comput Hum Behav. (2015) 52:49–58. doi: 10.1016/j.chb.2015.05.029

[16] Dong H Yang F Lu X Hao W. Internet addiction and related psychological factors among children and adolescents in China during the coronavirus disease 2019 (COVID-19) epidemic. Front Psych. (2020) 11:751. doi: 10.3389/fpsyt.2020.00751, PMID: 32982806 PMC7492537

[17] Chen S Weng L Su Y Wu H Yang P. Study on the development and psychometric characteristics of Chinese internet addiction scale. Chin J Psychol. (2003) 45:279–94. doi: 10.6129/CJP.2003.4503.05

[18] Huang L. An empirical study on the correlation between college students’ acculturation and e-learning (doctoral dissertation) Guangxi Normal University (2008).

[19] Hu LT Bentler PM. Cutoff criteria for fit indexes in covariance structure analysis: conventional criteria versus new alternatives. Struct Equ Model Multidiscip J. (1999) 6:1–55. doi: 10.1080/10705519909540118

[20] MacCallum RC Browne MW Cai L. Testing differences between nested covariance structure models: power analysis and null hypotheses. Psychol Methods. (2006) 11:19–35. doi: 10.1037/1082-989X.11.1.19, PMID: 16594765

[21] Czaja SJ Boot WR Charness N Rogers WA. Designing for older adults: principles and creative human factors approaches CRC press (2019).

[22] Hargittai E Hinnant A. Digital inequality: differences in young adults’ use of the internet. Commun Res. (2008) 35:602–21. doi: 10.1177/0093650208321782

[23] Van Laar E Van Deursen AJ Van Dijk JA De Haan J. The relation between 21st-century skills and digital skills: a systematic literature review. Comput Hum Behav. (2017) 72:577–88. doi: 10.1016/j.chb.2017.03.010

[24] Li N Kirkup G. Gender and cultural differences in internet use: a study of China and the UK. Comput Educ. (2007) 48:301–17. doi: 10.1016/j.compedu.2005.01.007

[25] Tian F Wang L. Research on the status of internet skills literacy of Chinese teenagers. Chinese Youth Soc Sci. (2020) 39:74–84. doi: 10.16034/j.cnki.10-1318/c.2020.06.011

[26] Chou C Wu H-C Chen C-H. Re-visiting college students’ attitudes toward the internet-based on a 6-T model: gender and grade level difference. Comput Educ. (2011) 56:939–47. doi: 10.1016/j.compedu.2010.11.004

[27] Hargittai E Hsieh YP. Succinct survey measures of web-use skills. Soc Sci Comput Rev. (2012) 30:95–107. doi: 10.1177/0894439310397146

[28] Qazi A Hasan N Abayomi-Alli O Hardaker G Scherer R Sarker Y. Gender differences in information and communication technology use & skills: a systematic review and meta-analysis. Educ Inf Technol. (2022) 27:4225–58. doi: 10.1007/s10639-021-10775-x PMC8528947 34697533

[29] Ameen N Tarhini A Shah MH Nusair K. A cross cultural study of gender differences in omnichannel retailing contexts. J Retail Consum Serv. (2021) 58:102265. doi: 10.1016/j.jretconser.2020.102265

[30] Shaouf A Altaqqi O. The impact of gender differences on adoption of information technology and related responses: a review. Int J Manag Appl Res. (2018) 5:22–41. doi: 10.18646/2056.51.18-003

[31] Teo TS Lim VK. Gender differences in internet usage and task preferences. Behav Inform Technol. (2000) 19:283–95. doi: 10.1080/01449290050086390

[32] Wang J Hong J-Z Pi Z-L. Cross-cultural adaptation: the impact of online social support and the role of gender. Soc Behav Personal Int J. (2015) 43:111–21. doi: 10.2224/sbp.2015.43.1.111

[33] Chen JJ Bai J. Internet use and academic achievement among Chinese adolescents: examining the mediating role of future orientation in a rural-urban dual system. Psychol Res Behav Manag. (2022) 15:2439–48. doi: 10.2147/PRBM.S343199, PMID: 36093412 PMC9462386

[34] Li Y Ranieri M. Are ‘digital natives’ really digitally competent? – a study on Chinese teenagers. Br J Educ Technol. (2010) 41:1029–42. doi: 10.1111/j.1467-8535.2009.01053.x

[35] Zhao H Ji A Jin C. The relationship between family functioning and adolescents’ online adjustment: the dual moderating effects of peer relationship and age. Psychol Tech Appl. (2022) 10:596–606.

[36] Andreassen CS Billieux J Griffiths MD Kuss DJ Demetrovics Z Mazzoni E. The relationship between addictive use of social media and video games and symptoms of psychiatric disorders: a large-scale cross-sectional study. Psychol Addict Behav. (2016) 30:252. doi: 10.1037/adb0000160 26999354

[37] Cao Y Rong D Zhao X Chen L Wang Q Cao Y. Analysis of health literacy and influencing factors of college students in Guiyang City. Chinese School Doctor. (2021) 7:1. doi: 10.4236/oalib.1107073

[38] Lin J Zhang J Zhao L. Survey of internet addiction among primary and secondary school students in Jiaxing City. Prophylactic Med. (2022) 34:1207–11. doi: 10.19485/j.cnki.issn2096-5087.2022.12.004

[39] Cerniglia L Griffiths MD Cimino S De Palo V Monacis L Sinatra M. A latent profile approach for the study of internet gaming disorder, social media addiction, and psychopathology in a normative sample of adolescents. Psychol Res Behav Manag. (2019) 12:651–9. doi: 10.2147/PRBM.S211873, PMID: 31496849 PMC6698172

[40] Chou C Hsiao M-C. Internet addiction, usage, gratification, and pleasure experience: the Taiwan college students’ case. Comput Educ. (2000) 35:65–80. doi: 10.1016/S0360-1315(00)00019-1

[41] Stanciu D Calugar A. What is irrational in fearing to miss out on being online. An application of the I-PACE model regarding the role of maladaptive cognitions in problematic internet use. Comput Hum Behav. (2022) 135:107365. doi: 10.1016/j.chb.2022.107365

[42] Kircaburun K Griffiths MD. Instagram addiction and the big five of personality: the mediating role of self-liking. J Behav Addict. (2018) 7:158–70. doi: 10.1556/2006.7.2018.15 29461086 PMC6035031

[43] Mai Y Hu J Yan Z Zhen S Wang S Zhang W. Structure and function of maladaptive cognitions in pathological internet use among Chinese adolescents. Comput Hum Behav. (2012) 28:2376–86. doi: 10.1016/j.chb.2012.07.009

[44] Tian Y Li W Guo J Yue W Chen P Li Y. Longitudinal associations among cumulative ecological risk, maladaptive cognitions and smartphone addiction in Chinese university freshmen: a two-wave study. Comput Hum Behav. (2023) 149:107921. doi: 10.1016/j.chb.2022.107552

[45] Kardefelt-Winther D. A conceptual and methodological critique of internet addiction research: towards a model of compensatory internet use. Comput Hum Behav. (2014) 31:351–4. doi: 10.1016/j.chb.2013.10.059

[46] Guerrini Usubini A Terrone G Varallo G Cattivelli R Plazzi G. The mediating role of emotion dysregulation and problematic internet use in the relationship between negative affect and excessive daytime sleepiness: a structural equation model. Nature Sci Sleep. (2022) 14:291. doi: 10.2147/NSS.S346485, PMID: 35237080 PMC8885123

[47] Goffman E. The presentation of self in everyday life. Oxford and Carlton: Blackwell Publishing (2007).

[48] Nie NH Hillygus DS. The impact of internet use on sociability: time-diary findings. IT Society. (2002) 1:1–20.

[49] Purwaningsih E Nurmala I. The impact of online game addiction on adolescent mental health: a systematic review and meta-analysis. J Med Sci. (2021) 9:260–74. doi: 10.3889/oamjms.2021.6234

[50] Ellis WE Dumas TM Forbes LM. Physically isolated but socially connected: psychological adjustment and stress among adolescents during the initial COVID-19 crisis. Can J Behaviour Sci. (2020) 52:177. doi: 10.1037/cbs0000215

[51] Folkman S. (2013). Stress: appraisal and coping. Gellman M. D. Turner J. R. (Eds) Encyclopedia of behavioral medicine. Springer, New York, NY

[52] Festinger L. A theory of social comparison processes. Hum Relat. (1954) 7:117–40. doi: 10.1177/001872675400700202

[53] Kharlamova DA Erofeeva MA. On the issue of formation of behavior of adolescents on the internet. Eur Proc Soc Behav Sci. (2022) 128:351–5. doi: 10.15405/epsbs.2022.11.49

[54] Baumeister RF Leary MR. The need to belong: desire for interpersonal attachments as a fundamental human motivation. Psychol Bull. (1995) 117:497. doi: 10.1037/0033-2909.117.3.497, PMID: 7777651

[55] Chou C Condron L Belland JC. A review of the research on internet addiction. Educ Psychol Rev. (2005) 17:363–88. doi: 10.1007/s10648-005-8138-1

[56] Ostovar S Bagheri R Griffiths MD Mohd Hashima IH. Internet addiction and maladaptive schemas: the potential role of disconnection/rejection and impaired autonomy/performance. Clin Psychol Psychother. (2021) 28:1509–24. doi: 10.1002/cpp.2581, PMID: 33687117

[57] Bozoglan B Demirer V Sahin I. Loneliness, self-esteem, and life satisfaction as predictors of internet addiction: a cross-sectional study among Turkish university students. Scand J Psychol. (2013) 54:313–9. doi: 10.1111/sjop.12049, PMID: 23577670

[58] Garland EL. Mindful positive emotion regulation as a treatment for addiction: from hedonic pleasure to self-transcendent meaning. Curr Opin Behav Sci. (2021) 39:168–77. doi: 10.1016/j.cobeha.2021.03.019, PMID: 34084873 PMC8168946

[59] Kates AW Wu H Coryn CL. The effects of mobile phone use on academic performance: a meta-analysis. Comput Educ. (2018) 127:107–12. doi: 10.1016/j.compedu.2018.08.012

[60] Longstreet P Brooks S. Life satisfaction: a key to managing internet & social media addiction. Technol Soc. (2017) 50:73–7. doi: 10.1016/j.techsoc.2017.05.003

[61] Shek DT Tang VM Lo C. Internet addiction in Chinese adolescents in Hong Kong: assessment, profiles, and psychosocial correlates. Scientific World J. (2008) 8:776–87. doi: 10.1100/tsw.2008.104, PMID: 18690381 PMC5848730

[62] Shek DT Tang VM Lo C. Evaluation of an internet addiction treatment program for Chinese adolescents in Hong Kong. Adolescence. (2009) 44:359–73. PMID: 19764272

[63] Tan Y Chen Y Lu Y Li L. Exploring associations between problematic internet use, depressive symptoms and sleep disturbance among southern Chinese adolescents. Int J Environ Res Public Health. (2016) 13:313. doi: 10.3390/ijerph13030313, PMID: 26985900 PMC4808976

