# Corrigendum: The influence of adolescents’ internet adaptation on internet addiction: the mediating role of internet cultural adaptation

**DOI:** 10.3389/fpsyt.2024.1415766

**Published:** 2024-05-21

**Authors:** Yue Yang, Jun Zhan, Yao Ni, Yanwen Fan, Yiting Zhang, Yiting Fang

**Affiliations:** ^1^ School of Psychology, Zhejiang Normal University, Jinhua, China; ^2^ Zhejiang Philosophy and Social Science Laboratory for the Mental Health and Crisis Intervention of Children and Adolescents, Zhejiang Normal University, Jinhua, China; ^3^ Postdoctoral Station of Psychology, School of Psychology, Fujian Normal University, Fuzhou, China; ^4^ Fujian Agriculture and Forestry University, Fuzhou, China

**Keywords:** internet addiction, adolescent internet use, internet cultural adaptation, internet adaptation, cross-cultural adaptation theory

In the published article, there was an error in the **Author List**. The first names and surnames of all authors had been reversed. The correct format is: ‘Yue Yang, Jun Zhan, Yao Ni, Yanwen Fan, Yiting Zhang and Yiting Fang’.

In the published article, there was an error regarding the **Affiliations** for authors Yiting Fang and Yiting Zhang. Instead of “^3^Postdoctoral Station of Psychology, School of Psychology, Fujian Normal University, Fuzhou, China” it should be “^4^Fujian Agriculture and Forestry University, Fuzhou, China”.

The authors apologize for these errors and state that this does not change the scientific conclusions of the article in any way. The original article has been updated.

